# Unveiling invisible farm‐to‐farm PRRSV‐2 transmission links and routes through transmission tree and network analysis

**DOI:** 10.1111/eva.13596

**Published:** 2023-09-15

**Authors:** Nakarin Pamornchainavakul, Dennis N. Makau, Igor A. D. Paploski, Cesar A. Corzo, Kimberly VanderWaal

**Affiliations:** ^1^ University of Minnesota Saint Paul Minnesota USA

**Keywords:** disease transmission, molecular epidemiology, network analysis, porcine reproductive and respiratory syndrome virus 2, reproduction number, traceability, transmission tree

## Abstract

The United States (U.S.) swine industry has struggled to control porcine reproductive and respiratory syndrome (PRRS) for decades, yet the causative virus, PRRSV‐2, continues to circulate and rapidly diverges into new variants. In the swine industry, the farm is typically the epidemiological unit for monitoring, prevention, and control; breaking transmission among farms is a critical step in containing disease spread. Despite this, our understanding of farm transmission still is inadequate, precluding the development of tailored control strategies. Therefore, our objective was to infer farm‐to‐farm transmission links, estimate farm‐level transmissibility as defined by reproduction numbers (*R*), and identify associated risk factors for transmission using PRRSV‐2 open reading frame 5 (ORF5) gene sequences, animal movement records, and other data from farms in a swine‐dense region of the U.S. from 2014 to 2017. Timed phylogenetic and transmission tree analyses were performed on three sets of sequences (*n* = 206) from 144 farms that represented the three largest genetic variants of the virus in the study area. The length of inferred pig‐to‐pig infection chains that corresponded to pairs of farms connected via direct animal movement was used as a threshold value for identifying other feasible transmission links between farms; these links were then transformed into farm‐to‐farm transmission networks and calculated farm‐level *R*‐values. The median farm‐level R was one (IQR = 1–2), whereas the *R* value of 28% of farms was more than one. Exponential random graph models were then used to evaluate the influence of farm attributes and/or farm relationships on the occurrence of farm‐to‐farm transmission links. These models showed that, even though most transmission events cannot be directly explained by animal movement, movement was strongly associated with transmission. This study demonstrates how integrative techniques may improve disease traceability in a data‐rich era by providing a clearer picture of regional disease transmission.

## 1 INTRODUCTION

In the swine industry, porcine reproductive and respiratory syndrome (PRRS) is a largely unmitigated disease and is one of the most common pig diseases globally (Lunney et al., 2010). The disease was named for its syndromic clinical manifestation that affect a wide range of individual and herd health metrics, including reproductive failure in sows measured by infertility, abortion, stillbirths, or giving birth to infected piglets, and respiratory disease in growing pigs that results in stunting and sometimes high mortality (OIE, 2008). Impacts on both breeding and finishing‐pig herds result in massive productivity losses estimated to be $664 million USD annually in the U.S. in 2012 (Holtkamp et al., 2013). Porcine reproductive and respiratory syndrome virus‐type 2 (PRRSV‐2) is the dominant causative agent of PRRS in North America (Shi et al., 2010). It is an enveloped positive‐stranded RNA virus assigned to the order *Nidovirale*s (Adams et al., 2016), family *Arteriviridae*, and species *Betaarterivirus suid 2* (Walker et al., 2021).

The RNA genome of PRRSV‐2 comprises several open reading frames (ORFs) that can be split into 3 major parts by their function: ORF1a and ORF1b encoding nonstructural proteins that modulate genome expression and replication, and 3′‐end ORFs encoding nucleocapsid and envelope proteins that are related to virus‐host interaction (Saberi et al., 2018; Snijder et al., 2013). Glycoprotein 5 (GP5), encoded by the ORF5 coding region, is one of the major envelope proteins (Gorbalenya et al., 2006) involved in in vivo neutralization (Wissink et al., 2003), infectious virion assembly (Wissink et al., 2005), and host cell entry (Delputte & Nauwynck, 2004). Many studies have found that ORF5 evolves under diversifying selection (Chen et al., 2016; Costers et al., 2010; Hanada et al., 2005; Storgaard et al., 1999), leading to high genetic variation which is suitable for a variety of phylogenetic analysis such as sub‐type classification (Paploski et al., 2019; Shi et al., 2010) and molecular epidemiology (Alkhamis et al., 2017). Thus, ORF5 is the most common gene that is sequenced and deposited to either public or private nucleotide sequence databases.

The virus is not only transmitted by direct contact between pigs but also indirectly through contact with contaminated fomites, iatrogenic farm practices, or aerosols (Pileri & Mateu, 2016), all of which may contribute to the spread of the virus between farms. Transport of PRRSV‐positive semen or animals is also mechanism for introducing the virus to other herds over long distances (Nathues et al., 2016; Thakur et al., 2015). In addition, contaminated trucks, equipment, or personnel sharing can transmit the disease via indirect contact (Dee et al., 2002; Dee et al., 2004). At short distances, PRRSV is possibly transmitted through aerosols based on experimental/semi‐experimental studies and air sampling near infected farms, though field evidence remains unclear (Arruda et al., 2019). Attempts to estimate the relative contributions of these routes to the overall PRRSV‐2 transmission have been made using a simulation model that utilized disease incidence and between‐farm contacts as an input (Galvis et al., 2021). However, exploitation of the genetic relatedness among viruses found on different farms may provide a clearer, empirical‐based picture of disease transmission (Firestone et al., 2019).

Based on experimental studies (Charpin et al., 2012; Rose et al., 2015) and mathematical modeling (Nodelijk et al., 2000), the European PRRSV genotype (PRRSV‐1) has an estimated R_0_ of 2–5, meaning that an average infected pig transmits the virus to 2–5 other pigs (assuming an immunologically naïve population). In contrast to R_0_, the effective reproduction number (R_e_, R_t_, or R) relaxes the assumption of the population being fully susceptible and is defined as the average number of secondary cases that are infected by a single infectious individual regardless of immune status of the population (Nishiura & Chowell, 2009). R is often used to measure disease transmissibility for endemic diseases or unfolding epidemics and, importantly, can be used to help quantify the impact of control measures. However, neither R_0_ nor R measured at animal level can explain between‐farm transmissibility, which drives PRRSV persistence at a regional scale and is the level at which control measures are implemented.

Using PRRSV‐2 genetic data coupled with information related to farm characteristics and contact between farms, our objective was to infer farm‐to‐farm transmission links, estimate farm‐level transmissibility as defined by reproduction numbers (R), and identify associated risk factors for transmission. We analyzed a set of PRRSV‐2 ORF5 sequences collected from swine farms along with animal movement data in a swine‐dense region of the United States (U.S.) to fill knowledge gaps on the between‐farm transmissibility and pathways of spread. Farm‐level R and potential pathways were estimated by integrating transmission tree inference of PRRSV‐2 sequences with network‐based statistical inference. Our results not only illuminate a clearer picture of PRRSV‐2 dynamics in a major swine‐producing region of the U.S. but also demonstrate a novel approach to quantify the between‐farm transmissibility of PRRSV‐2 that can be expanded to evaluate the effectiveness of control measures across space and time.

## 2 MATERIALS AND METHODS

### 2.1 Data selection

Most data used in this study were obtained from the Morrison Swine Health Monitoring Project (MSHMP) database, which was established to track progress on PRRS control in the U.S. and aimed to be the national hub for voluntary data sharing between swine veterinarians from different production systems (MSHMP, 2021). The project has archived farm‐level data such as farm location, herd size, disease incidence, and pathogen genetic sequences from more than half of the U.S. breeding population (Paploski et al., 2019). For this particular study, we focused on a major swine‐dense farming region (confidential data) in the U.S. in which 70% of farms (*n* = 2724) belong to two multi‐site swine production systems that participate in MSHMP. A swine production system is a commercial entity consisting of multiple swine production sites connected by either ownership, management, or contractual agreements (Kinsley et al., 2019; Makau, Paploski, & VanderWaal, 2021). Production systems are very insular, with nearly 100% of animal movements occurring between farms within the same system (Kinsley et al., 2019).

Phylogenetic analysis of PRRSV‐2 ORF5 sequences has been used for virus lineage and sub‐lineage classification (Paploski et al., 2019; Shi et al., 2010). Based on the frequency of samples submitted to MSHMP, lineage 1A (L1A) was found to be a predominant PRRSV‐2 sub‐lineage in the U.S. since 2014 (Paploski et al., 2019). Moreover, a previous study also suggested that the L1A virus was either introduced to or emerged in our study area in early 2013 and started expanding within the region in 2014 (Makau, Alkhamis, et al., 2021). Focusing our analysis on the emergence and spread of L1A viruses from 2014 to 2017, we queried 1515 voluntarily submitted ORF5 sequences from the study area that were identified as L1A in the MSHMP database. Field veterinarians typically requested ORF5 sequencing for routine diagnosis during a PRRS outbreak (particularly at the beginning) after case confirmation by RT‐PCR. The sequences were aligned using MAFFT (Katoh, 2002) and screened for potential recombination using RDP4 (Martin et al., 2015). Subsequently, a maximum likelihood phylogeny was reconstructed from the alignment using RAxML (Stamatakis, 2014) with the GTRCAT nucleotide substitution model and transfer bootstrap clade support computation (Lemoine et al., 2018) from 1000 bootstrapped trees. To prevent potential bias caused from multiple sequences available from the same farm, sequences from a single farm that formed monophyletic clades on the tree were subsampled, retaining the median‐dated sequence. The filtered dataset contained 943 sequences derived from 651 farms between 2014 and 2017.

**FIGURE 1:**
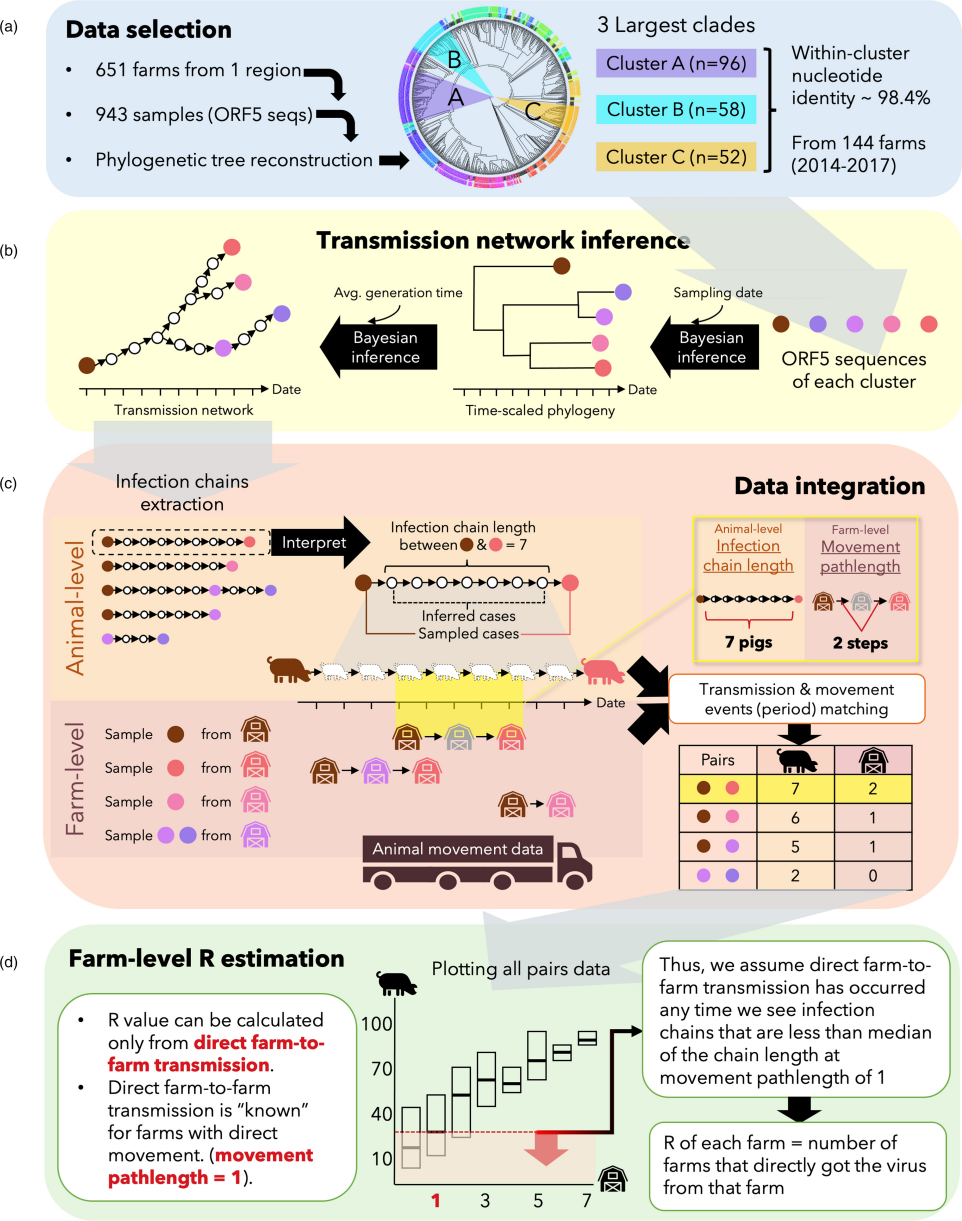
Workflow of farm‐to‐farm network reconstruction and R estimation. The three biggest phylogenetic clusters of the lineage 1A PRRSV‐2 were selected for the analysis (a). Each cluster's ORF5 sequences were used to reconstruct the time‐resolved phylogenetic tree and transmission tree (b). Pig‐to‐pig infection chains were extracted from the transmission tree and then matched with animal movement data (c). Infection chain length was used to estimate direct farm‐to‐farm transmission links that were combined into a farm‐level transmission network. Farm‐level effective reproduction number (R) was calculated from the network (d).

In order to focus the analysis on groups of sequences that were more likely to be epidemiologically linked, we identified the largest three clusters of closely related sequences from the phylogenetic tree, then conducted further analyses for each cluster separately (Figure 1a). Groups of monophyletic sequences were systematically defined as a cluster with Cluster Picker (Ragonnet‐Cronin et al., 2013) when their bootstrapped clade support was >70% and the maximum genetic distances within the group was <4.5%.

The animal movement data used in this study were directly obtained from the two participating production systems, which electronically recorded all farm‐to‐farm movements of pigs, totaling 283,959 movement events among 2724 farms during 2014–2017. Premise ID, geographic coordinates, farm production types of the origin and destination farms, and shipment date of each event were prepared for the network analysis. Production types included sow farm (14.7%), nursery (17.5%), finisher (67.1%), and boar stud (0.7%). A sow farm is a premise that comprises at least breeding and farrowing sows or gilts population. A nursery farm is a premise that only raises pigs from newly weaned to grower stage. A finisher farm is a premise that feeds grower pigs until they reach the market weight. A boar stud is a premise where boars are raised for semen collection.

### 2.2 Transmission network inference

Phylogenetic temporal signals of the three clusters were checked from maximum likelihood sub‐trees using TempEst (Rambaut et al., 2016); *R* ^2^ and correlation coefficient above 0.5 and 0.4 respectively were considered to be evidence of sufficient temporal signal in the data for construction of time‐scaled phylogenies. We constructed time‐scaled phylogenies for the clusters in BEAST 1.10.4 (Drummond & Rambaut, 2007) using a Monte‐Carlo Markov Chain (MCMC) length of 10 million. The cluster's ORF5 alignment and sampling date were inputted and run with the GTR + I + G substitution model selected based on Bayesian information criterion computed by ModelFinder (Kalyaanamoorthy et al., 2017), the uncorrelated relaxed clock model (Drummond et al., 2006), and the time‐aware Bayesian skyride model (Minin et al., 2008). Time‐scaled phylogenetic trees were constructed by the maximum clade credibility (MCC) method, excluding the first million burn‐in MCMC states using TreeAnnotator 1.10.4 (Drummond & Rambaut, 2007). To infer transmission networks from the MCC trees, we conducted transmission tree analysis in *TransPhylo* 1.4.5 in R (R Core Team, 2019). Among several available packages for transmission tree inference, TransPhylo had the most appropriate assumptions and options that best aligned with our data, notably in that it allows for incomplete sampling of cases and ongoing (endemic) outbreak scenarios (Didelot et al., 2017; Duault et al., 2022). Given a time‐scaled phylogeny, molecular clock, and assuming a stochastic branching epidemiological process, TransPhylo uses a Bayesian approach to create a network indicating who infected whom and inferring the number of unsampled individuals in the transmission chain connecting a pair of sampled cases (Didelot et al., 2017). To parameterize the model priors, we assumed that the observed transmission processes were part of an ongoing outbreak (i.e., the outbreak was not resolved by the last sampling point) and that the PRRS mean generation time was 14.5 days (Charpin et al., 2012). The analyses were executed with 50,000 MCMC iterations (Figure 1b).

The outputs of the transmission tree analysis include inferred pig‐to‐pig transmission chains, with information on the origin, recipient, and infection window coinciding with the sampling date on the first and second sequence. These outputs can be conceptualized as a dynamic directed network. Since the direction of transmission at the animal level may or may not reflect directionality at the farm level, we transformed the trees into undirected networks and then used the igraph package (Csardi & Nepusz, 2006) to compute the shortest pathlength (SPL) between all the sampled pairs, disregarding directionality, to capture the most feasible pig‐to‐pig infection chain between each pair of samples. Although TransPhylo can take into account within‐host evolutionary dynamics, this was not implemented in our model given that the scale of variation occurring at the between‐farm level likely is far greater than variation arising from within‐host evolution. Also, our infection chains (at the pig level) were not sufficiently well sampled to be able to discern within‐host evolutionary processes.

### 2.3 Data integration

In order to translate the transmission network from the animal level to the farm level, we used the farm ID associated with each sequence to create a farm‐level transmission network, assuming that the virus must have moved between farms somewhere along the pig‐to‐pig infection chain inferred in the transmission network analysis. However, direct farm‐to‐farm transmission cannot be assumed between farms connected in the transmission network, as it is possible that the infection chain has passed through an unsampled intermediate farm. Therefore, we used animal movement data to identify “known” direct transmission events, wherein direct farm‐to‐farm transmission can be reasonably assumed if an animal movement occurred between the farms during the infection window. While there are multiple modes of between‐farm contact that could lead to transmission between farms, pairs of farms connected via animal movement are a form of contact for which we have quantifiable data on direct contact between farms. We used the infection chain lengths of inferred transmission events that were concurrent with documented between‐farm animal movement to define a maximum threshold in the length of pig‐to‐pig infection chains, below which direct transmission between farms (regardless of the presence of a movement) is feasible. To do this, we created a time‐stamped dynamic movement network and extracted the shortest path between the samples existing during the infection window (sample dates of the earlier and later sequences collected from two different farms) of every sampled pair. Between each pair of samples, the infection chain length (ICL) was designated as the inferred number of unsampled pigs in the shortest path connecting each pair in the transmission network, and the movement pathlength (MPL) was designated as the number of steps (i.e., farms) that must be passed through to connect two farms in the movement network (Figure 1c). Two farms were considered “unreachable” if no path existed that connected the two farms during the infection window. Viable paths in the movement network must follow the directionality and the temporal sequence in which movements occurred. Pearson's correlation coefficient between ICL and MPL of each cluster was calculated. Since the farm‐level R can be calculated only from direct farm‐to‐farm transmission, we assumed that direct farm‐to‐farm transmission had occurred in any case where the infection chain was shorter than the median of ICL at MPL of 1 (regardless of the presence of documented animal movement) and used the resulting farm‐to‐farm transmission events to create a farm‐level transmission network.

Considering uncertainties in the PRRSV generation time used to infer animal‐level transmission and the length of time prior to sample collection that PRRSV‐2 could be circulating in a farm, we performed two sensitivity analyses. First, we re‐specified the generation time to 11.6 and 17.4 days (plus and minus 20% from the originally estimated 14.5 days). Second, we extended the infection window start date to 3 and 6 months before the original window. This allows the farm to be infected earlier than the sample was collected. We then repeated all procedures for each setting.

### 2.4 Farm‐level R estimation

All the farm‐representative sample pairs that had infection chain lengths less than the threshold (median of ICL at MPL of 1) and sampling date interval less than 1 year were considered candidate transmission pairs for R estimation. In some cases, one recipient farm may have multiple potential sources of transmission present in the candidate list. Thus, for each recipient, we selected the single source with the shortest ICL as the most probable. Farm‐level R values for different phylogenetic clusters and sensitivity analytic settings were then computed by counting the number of recipients per each source and summarized into descriptive statistics (Figure 1d). Details and code for our approaches are available at https://github.com/author's identifier/FarmR.

### 2.5 Analysis of potential factors associated with farm‐to‐farm PRRSV‐2 transmission

Farm‐level metadata, including location and production type, were utilized to investigate potential modes of transmission between farms beyond animal movement. The distance between a pair of farms was calculated from their geographical coordinates. The approximate longest distance that viable PRRSV‐2 can be found in aerosols, 10 km (Arruda et al., 2019; Otake et al., 2010), was set as a threshold for classifying farms in close enough proximity for potential local‐area spread. PRRSV‐2 transmission through contaminated semen was presumed when a transmission pair contained boar‐sow farms as the source recipient. Altogether, the mode of transmission for pairs that were not connected via animal movement was designated into the following nonmutually exclusive categories: farm proximity‐related factor, transmission by contaminated semen, and undetermined. Undetermined may include a wide variety of transmission modes for which we do not have data, such as movement of equipment, feed, and personnel.

Farm‐to‐farm transmission pairs used for the R value calculation were transformed into directed transmission networks for each cluster. We applied multivariable exponential random graph models (ERGMs) to the networks to identify factors significantly associated with the occurrence of an inferred transmission link using the ergm 4.3.2 package in R (Hunter et al., 2008). ERGMs are a type of statistical regression that treat network topology as a response and edge/node attributes as predictors. The output of ERGMs includes the odds that a particular attribute influences the network structure. In our case, the analysis indicates which factor is significantly associated with the existence of a transmission link between two farms. The model was initially constructed with the best‐fit structural covariate (in‐stars; frequency of star‐like network structures in which several nodes connect to the same central node without connection with each other) that accounted for the underlying architecture of the directed network. Put simply, this was a baseline model that captures basic characteristics of network structure but did not account for how node‐ or dyad‐level attributes influenced which nodes were connected.

Additional node‐ or edge‐level covariates were then added to the baseline model. Node‐level covariates included a farm's production type, herd size, season in which the sequence was sampled, farm density, and in‐ and out‐degree in the movement network. Sampling time was classified into season by month, i.e., winter (December to February), spring (March to May), summer (June to August), and fall (September to November). Farm density was summarized for a 10 km radius around each farm and was computed from regional pig farms' coordinates using the PointdensityP package (Evangelista & Beskow, 2019). In‐degree and out‐degree (number of farms that the focal farm received or sent animals to in a six‐month period) were computed using the igraph package (Csardi & Nepusz, 2006) from a 6‐month period that was temporally matched with the sample. Six months was selected based on previous work demonstrating that degree metrics calculated in swine movement networks reach stability when six months of data are aggregated (Makau, Paploski, & VanderWaal, 2021). These node/edge attributes were incorporated into the model using several different ERGM terms. Categorical node‐level covariates, such as production type and sampling season, were incorporated with the terms *nodefactor* (e.g., some production types are generally more likely to form transmission links), *nodematch* (e.g., transmission links are more likely to be found between nodes with the same production type), and *nodemix* (e.g., accounting for differential frequencies with which transmission links form between farms of different production types). The *absdiff* (e.g., two farms with similar herd size are more likely to have transmission link) and *nodecov* (e.g., farms in higher density areas are more likely to form transmission links) terms were used for continuous node‐level covariates.

Three edge‐level covariates were also included in the model: geodesic distance (km), MPL (steps in the movement network) between the sampled pair, and sampling date interval (days). We calculated geodesic distance (km) using the geosphere package in R (Hijmans et al., 2019) and then dichotomized using 10 km as a cut‐off value (0 = more than 10 km, 1 = less than 10 km). Based on the distribution of MPL, we assigned MPLs of ≤3 steps as possible movement connections (Lunney et al., 2010), while >3 as not (0). Sampling date interval was included to control for temporality in the model (i.e., samples collected close in time were more likely to have short infection chain lengths and be linked in the transmission networks). The *edgecov* term (e.g., transmission links are more likely found between farms that have animal movement connections) was used for both categorical or continuous edge‐level covariates.

We performed an AIC‐based stepwise approach to build multivariable ERGM models from all predictors. Coefficients (log‐odds), probabilities (inverse logit), and p‐values of predictors were reported from the most parsimonious model that was <2 delta‐AIC from the model with the lowest AIC.

## 3 RESULTS

### 3.1 Descriptive analysis and phylodynamics

**FIGURE 2:**
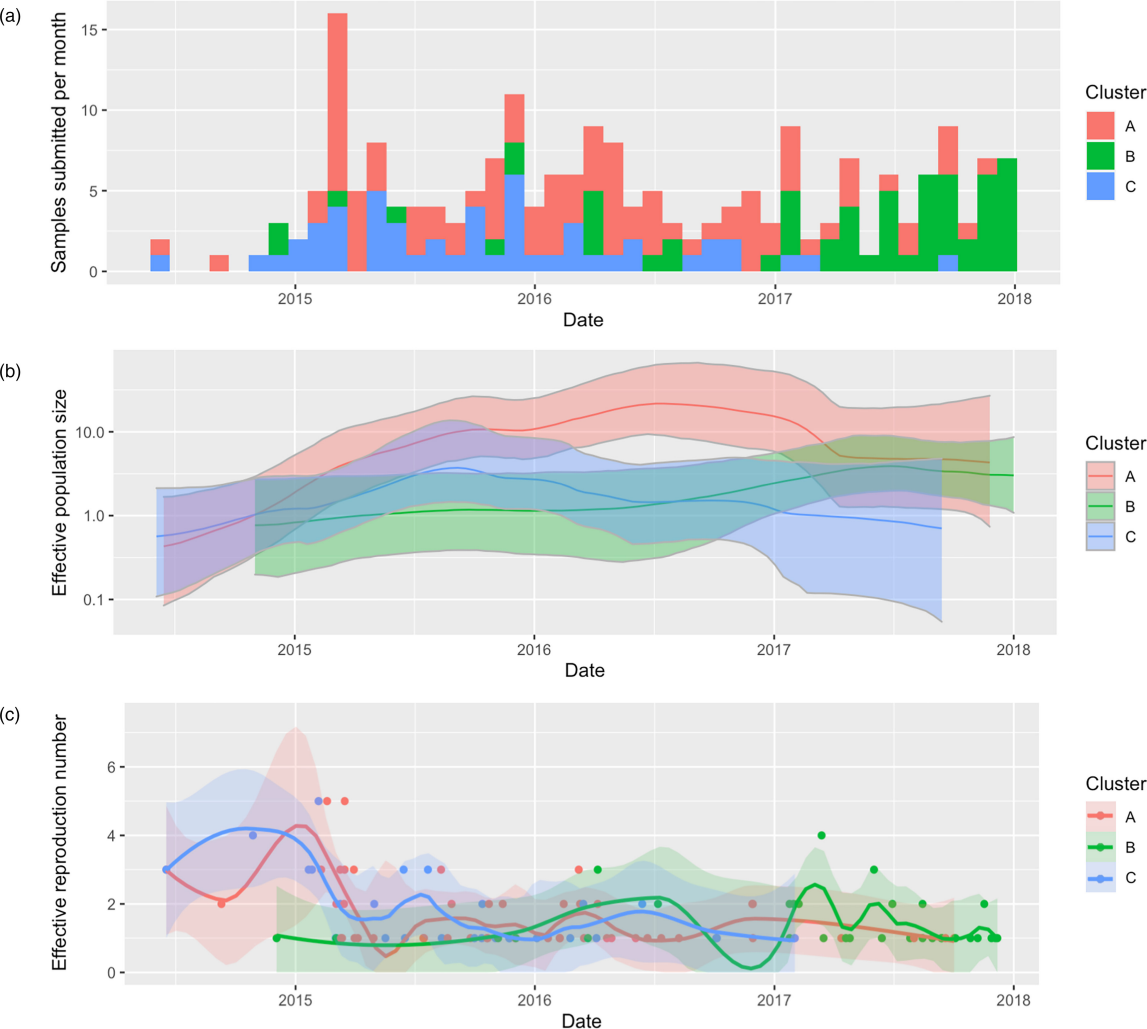
PRRSV‐2 dynamics of the three genetic clusters during 2014–2017. Number of ORF5 sequences submitted per month (a). Median effective viral population size with the 95% highest posterior density (HPD) estimated by Bayesian skyride analysis (b). Scatterplot of the effective reproductive number of individual farms, dated according to source farm's sampling date, with LOESS curves overlayed to visualize temporal trends (c).

The three largest phylogenetic clusters of PRRSV‐2 lineage 1A, denoted as cluster A (*n* = 96 sequences), B (*n* = 58 sequences), and C (*n* = 52 sequences), were included in the analyses (Table 1). All three clusters contained sequences identified from farms that all belonged to a single production system. Pairwise genetic distance within each cluster was 1.6%–1.8%, and the distance between clusters was >2.6%. An average of ~1.4 (SD: 0.85) ORF5 sequences was available from each farm that were part of clusters A–C. More than half of the samples were collected from sow herds, followed by nursery, finisher, and boar farms (Table 1). In three cases, serially collected samples from three farms were classified into different clusters. Maximum likelihood trees constructed for each cluster exhibited a strong temporal signal indicated by high correlation coefficients between root‐to‐tip divergence and tip date, ranging from 0.73 to 0.79 with acceptable *R* ^2^ values (0.53–0.63). Based on inference from Bayesian time‐scaled phylogenies, mean viral evolutionary rates were relatively consistent across three clusters (7.2–9.8 × 10^−3^ substitutions/site/year) and the time to the most recent common ancestors (tMRCA) for each cluster was in early 2014 (Table 1). The Bayesian Skyride analysis suggests that the effective viral population of clusters A and C sharply increased from mid‐2014 until early 2015, then cluster C's population decreased after summer 2015, whereas cluster A plateaued and decreased in late 2016. The effective population of Cluster B was originally the smallest, but gradually rose starting in early 2016 and was comparable to cluster A in late 2017 (Figure 2b).

**TABLE 1:** Data structure, genetic relationship, temporal signal of the selected clusters' ORF5 sequence samples, and key statistics from their time‐resolved phylogenetic trees.

		Cluster A	Cluster B	Cluster C
Data description	Number of samples	96	58	52
	Average pairwise identity (%)	98.2	98.4	98.5
	Bootstrap support at ancestral node (%)	86.3	93.9	71.1
	Sampling date range (dd/MM/yyyy)	18/6/2014–28/11/2017	3/12/2014–29/12/2017	18/6/2014–27/9/2017
	Number of farms	72	42	36
	Number of sow farms (%)	41 (56.9%)^a^	22 (52.4%)^a^	18 (50%)
	Number of nursery farms (%)	16 (22.2%)^b^	15 (35.7%)^b^	9 (25%)
	Number of finishing farms (%)	13 (18.1%)	5 (11.9%)	7 (19.4%)
	Number of boar studs (%)	2 (2.8%)	0 (0%)	0 (0%)
	Number of unidentified farms (%)	0 (0%)	0 (0%)	2 (5.6%)
Temporal signal (Root‐to‐tip divergence ~ time)	Correlation coefficient	0.78	0.73	0.79
	*R* ^2^	0.61	0.53	0.63
Bayesian timed phylogeny estimation	Mean Rate (substitutions/site/year)	7.20 × 10^−3^	7.25 × 10^−3^	9.82 × 10^−3^
	Mean tMRCA	2014.3	2014.5	2014.2
	95% HPD interval	[2014.0, 2014.5]	[2014.1, 2014.8]	[2013.9, 2014.5]

^a^ Two sow farms submitted samples belonging to different clusters (A in 2015–2016 and B in 2017).

^b^ A nursery farm submitted samples belonging to different clusters (A in 2016–2017 and B in 2017).

### 3.2 Inferred infection chains and animal movements

Transmission tree analysis estimates the number of unsampled cases between a pair of samples, referred to as the infection chain length (ICL). Movement pathlength (MPL) is the shortest number of animal movement steps that corresponds to the sampled farms in the infection chain during the infection window (dates of sample A and B). Linear regression shows that ICL and MPL, combined across all clusters, were significantly correlated (*R* ^2^ = 0.29, Pearson's *r* = 0.53, *p* < 0.001), indicating that pairs of farms that were more distant to each other in the movement network also had longer inferred infection chains. According to the sensitivity analysis that varied the assumed mean generation time and infection window length, this correlation was robust to uncertainties in the generation time and to extending the infection window used for calculating movement pathlengths (Figure S1). Given that movement pathlengths of 1 represent “known” direct contact between farms, we assumed that direct farm‐to‐farm transmission was reasonably likely for any pair of farms where the number of pigs in an infection chain was less than median of ICL at MPL of 1. This threshold was applied to identify candidate transmission pairs across all pairs (regardless of presence of movement). Accordingly, the threshold ICL for farm‐to‐farm transmission for clusters A, B, and C were 35, 28, and 46 pigs, respectively.

### 3.3 Farm‐level R and transmission events

We were able to infer 80, 45, and 49 farm‐to‐farm transmission events in cluster A, B, and C, respectively. Farm‐level effective reproduction numbers (*R*) were computed for each source farm that appeared in the list of candidate transmission pairs (events). Across all clusters, R had a median of 1, with interquartile ranges (IQR) of 1–2 for cluster A and B, and 1–2.5 for cluster C. This indicates that an infected farm typically infects 1 to 2 additional farms. The number of farms having *R* > 1 in cluster A, B, and C was 20, 10, and 12, respectively. The highest observed Rs for cluster A and C (*R* = 5) were all observed between February to March 2015, whereas the cluster B's highest farm‐level R (*R* = 4) was observed in March 2017 (Figure 2c). The median *R* did not change when the generation time and infection window for capturing time‐matched movements were varied, with the exception of one scenario for cluster C (generation time of 17.4 days and 3–6 months relaxed timeframes) for which the median *R* was 2 (Figure S3).

**FIGURE 3:**
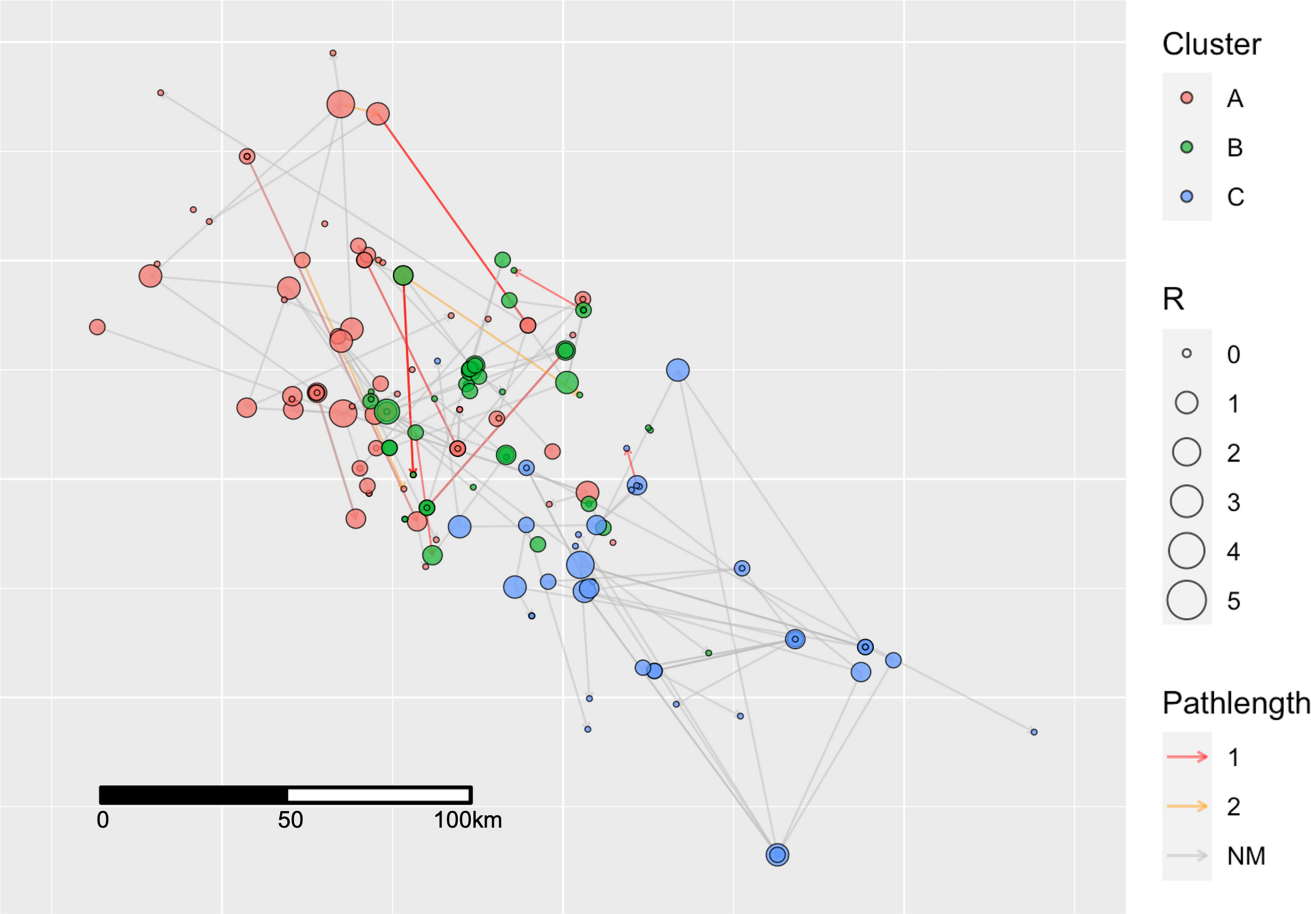
Spatial representation of the estimated transmission network of farm‐to‐farm PRRSV‐2 transmission from 2014 to 2017. Node color represents designated phylogenetic clusters (A–C). Node diameter size corresponds to an individual farm's effective reproduction number (R). Edge color represents movement pathlengths (NM; No movement). Samples with unknown farm location (*n* = 6) were dropped from the network.

Overall, over 80% of the farm‐to‐farm transmission events had no corresponding animal movement linking the two farms. For the events that were not linked by animal movements, 8.3 to 23.7% of the transmission events across clusters involved farms located less than 10 km apart, whereas the longest inferred transmission range was over 100 km for every cluster (Figure 3). Most transmission events occurred between sow herds (32.7%) followed by sow‐to‐nursery (13.8%) and nursery‐to‐sow (12.6%). Relative to the directionality of pig production flows (pigs more from sow farms to nurseries to finishing farms), the direction of transmission could be downstream (71.3%) or upstream (28.7%). Interestingly, two boar studs in cluster A had relatively high *R* (2 and 3), and the recipients were four different sow farms. The source of infection to these boar studs appeared to be sow and finishing farms.

### 3.4 Factors associated with transmission

We created directed networks from inferred farm‐to‐farm transmission events, then fit exponential random graph models (ERGMs) (Hunter et al., 2008) to identify and measure factors associated with transmission links (Tables S1–S3). Clusters A and B's networks were explained by the same best‐fit model, and Cluster C's model differed by a single variable (Table 2). Our best‐fit models suggest that farms within a 10 km radius of one another were > 10 times less likely to have a transmission link (odds = 0.03–0.1) than farms located more than 10 km apart. The odds of having a transmission link increased by 22 (cluster A) to 37 times (cluster B) if there were animal movements (MPL 1–3 steps) between two farms. The log‐odds of a transmission link decreased by 0–0.01 for each additional day of sampling date interval, meaning that the odds of two farms forming a transmission link was halved after ~70 days. Pairs of farms in areas with similar farm density had an increased log‐odds of 0.02 for cluster A and 0.04 for cluster B. Particularly for cluster A, transmission links were more likely (odds = 1.9) between farms whose samples were collected in different seasons (Table 2). Multicollinearity among the predictors was not detected in any model according to the ERGMs' variance inflation factor.

**TABLE 2:** ERGMs' predictors of each cluster's network with coefficients reported on the log‐odds (odds) scale.

	Cluster A			Cluster B			Cluster C		
	Coefficient	SD	*p*‐value	Coefficient	SD	*p*‐value	Coefficient	SD	*p*‐value
Structural term: In‐stars	−2.59 (0.08)	0.29	<0.001***	−2.10 (0.12)	0.45	<0.001***	−2.12 (0.12)	0.40	<0.001***
Animal movement (y/n)	3.10 (22.20)	0.41	<0.001***	3.62 (37.34)	0.71	<0.001***	1.42 (4.14)	0.79	0.074
Farm proximity <10 km (y/n)	−2.42 (0.09)	0.34	<0.001***	−2.27 (0.10)	0.45	<0.001***	−3.41 (0.03)	0.56	<0.001***
Difference in farm densities surrounding the source / recipient farms (continuous)	−0.02 (0.98)	0.01	0.029*	−0.04 (0.96)	0.01	0.004**	−0.02 (0.98)	0.01	0.055
Sampling date interval (days)	−0.01 (0.99)	0.00	<0.001***	−0.01 (0.99)	0.00	<0.001***	0.00 (1.00)	0.00	<0.001***
Same sampling season (y/n)	−0.66 (0.52)	0.33	0.049*	0.60 (1.82)	0.40	0.131			
Same Farm type (y/n)							0.55 (1.73)	0.34	0.104

## 4 DISCUSSION

In this paper, we implemented novel integrative approaches that utilized routinely collected PRRSV‐2 genetic sequences, animal movement records, and farm metadata to strengthen our ability to trace the spread of PRRSV‐2 between farms. We used this approach to infer farm‐to‐farm transmission links, quantify the farm‐to‐farm transmissibility of the virus by estimating farm‐level effective reproduction numbers, and identify factors associated with disease transmission. In summary, the analysis suggests that most infected farms transmitted the virus to one additional farm, though several potential super‐spreader events (*R* substantially above 1) were observed. Our *R* value estimation, however, likely underestimated the true spread due to potential shortcomings associated with sequencing data generation. Although most transmission events could not be attributed directly to animal movement, movement was a crucial risk factor associated with between‐farm transmission links. In addition, the odds of an inferred transmission link between two farms reduced by 50% by ~70 days after the sampling date (likely when the outbreak was recognized) at the source farm. This suggests that most onward transmission from farms occurs within the first two months or so.

Historically, restriction fragment length polymorphism (RFLP) typing, percent genetic distance comparisons, and phylogeographic reconstruction have been used to assess or track PRRSV spread in different situations (Alkhamis et al., 2017; Liu et al., 2021; Rosendal et al., 2014). The core principle of these analyses is an association between shared genotype or phylogenetic relatedness with other attributes of the farms that could help disentangle the likely route of transmission, such as measurable host contacts, spatial adjacencies, temporal continuity. Here, we further expand the concept by converting time‐scaled phylogenies into high‐resolution transmission networks that infer who infected whom and how many animals were potentially involved in the infection chain between a pair of samples (based on the generation time and molecular clock of the virus). The estimated evolutionary rates and trends in viral population growth of the observed clusters are consistent with analysis of PRRSV‐2 sub‐lineage L1A drawn from nationwide ORF5 databases (7.62–7.72 × 10^−3^ substitutions/site/year with the peak of population size in 2016) (Paploski et al., 2021). In addition, the detection of multiple clusters through time on individual farms emphasizes the possible role of re‐infection by closely related viral variants, which could potentially contribute to disease persistence at the local or regional scales.

Pig‐to‐pig transmission networks, however, cannot be interpreted in the same manner as person‐to‐person transmission for a human disease (Hatherell et al., 2016) since pig populations are highly discretized into relatively homogenous sub‐populations (i.e., farms). Indeed, between‐farm rather than between‐animal transmission is more important for understanding regional spread and pathogen persistence. To make sense of that, the animal‐level network was transformed into the farm‐level network, and time‐matched animal movement data was used to inform the maximum length of animal‐level infection chains that would be consistent with direct farm‐to‐farm transmission (as opposed to longer infection chains that may be more likely to have passed through an unsampled intermediate farm). Many of the inferred transmission pairs had no recorded connections via animal movement, highlighting the fact that other transmission modes play a role in between‐farm spread. We were limited by data availability on other transmission routes, and therefore, we assumed that the infection chain lengths for other modes of transmission would be similar to that of animal movement‐mediated transmission, which is a limitation to our approach.

Even though live animal movement within the study production system was well‐documented, most between‐farm transmission events could not be explained by movement. This phenomenon possibly emerges for three reasons. First, live animal movement prior to an outbreak (particularly movements from a PRRS‐positive farm) may be viewed as a “smoking gun” or a primary suspect for the route of disease introduction. In such cases, field veterinarians may not submit a sample for sequencing given that the source of outbreak appears obvious. Second, other undocumented transmission routes may be playing a substantial role in between‐farm transmission. For example, while boar studs are seldom infected with PRRS, sequences associated with two outbreaks at boar studs had higher than average farm‐level Rs. Sow farms were the recipients in all cases, likely suggesting transmission via contaminated semen. Other kinds of transport may also disseminate the virus, such as fomites transported by contaminated vehicles or equipment, between farms can contribute to long‐distance transmission (Dee et al., 2002; Dee et al., 2004). For example, our analysis suggests that the virus was transmitted from a positive nursery farm to a finishing farm (103 km apart) and then the recipient transmitted the virus back to the origin within a few months, but the matched movement event was only detected in the first event (nursery to finisher). The latter event is one of several inferred transmission links where the directionality is opposite to the unidirectional flow of animals through farms typical of a vertically integrated pig production system (Lee et al., 2017; Passafaro et al., 2020, Figure S4). That being said, we cannot precisely conclude the contribution of each mode of transmission without a complete sequence database for all farms and concrete data associated with other transmission modes, such as semen samples and delivery history, all between‐farm traffic records, or contemporary environmental samples.

Our results indicate that PRRS outbreaks have a farm‐level R of ~1. This finding is consistent with previous estimations of R using PRRS incidence data throughout the U.S. from 2009 to 2016 (Arruda, Alkhamis, et al., 2017), though a seasonal pattern of super‐spreader events (*R* > 1) was not clearly detected in our network. The timing of super‐spreader events, however, was concurrent with the increases in the effective population size shown by the phylodynamic analysis and in disease incidence depicted by frequency of sample submission (Figure 2a). Taken together, this suggests that expansions in regional transmission were at least partly coincident with super‐spreader events as opposed to multiple one‐to‐one transmission events. More generally, applying our integrative approach can enhance disease monitoring efforts by providing fine‐scale epidemiologic assessment such as estimating patterns of spread of PRRSV‐2 variants in a particular region or production system, or comparing between‐farm transmissibility before and after control interventions.

The sensitivity analysis showed that altering either the mean generation time or the timeframe for identifying time‐matched movements only slightly affected the infection chain length used as a threshold for direct transmission (Figure S2) and estimated farm‐level *R* values (Figure S3). This means, first, biological variation in animal‐level factors that may influence generation time, such as host contact rates and host‐pathogen interactions (Liu et al., 2018), may not substantially contribute to variation in farm‐level transmissibility. Second, while there was a concern that animal movements that occurred prior to sample collection on the farm may allow for transmission beyond the infection window, our results from sensitivity analysis were not substantially altered by extending this timeframe to include movements that had occurred 3–6 months earlier than the original infection window. This is potentially because animal movements between specific pairs of farms often recur at regular intervals through time (Makau, Paploski, & VanderWaal, 2021), such that few new and unique connections were identified by extending the infection window.

Possible between‐farm PRRSV transmission routes are well documented (Arruda et al., 2019; Dee et al., 2002; Dee et al., 2004; Nathues et al., 2016; Thakur et al., 2015), but their contribution to the regional disease endemicity is somewhat vague. Our results further point toward animal movement as an important but not sole mode of transmission. The ERGM analysis highlights the role of animal movement as a primary risk factor for transmission (although the coefficient for animal movement in Cluster C was only trending toward significance). Once animals were shipped from an infected farm, the shipping destination's risk of becoming infected is many folds higher than farms that do not receive animals from an infected source. We also hypothesized that local area spread of PRRSV‐2 (less than 10 km) might explain some transmission events which animal movement cannot. Surprisingly, the network analysis revealed that a short distance between the pair of farms was a protective factor for the occurrence of a transmission event between farms. Apart from mechanical transmission routes such as infectious fomites or personnel sharing among neighbors, this result suggests that between farm PRRSV‐2 transmission rarely occurs via the airborne route, which agrees with the conclusions of the previous reviews and phylogeographic analysis (Arruda et al., 2019; Makau, Alkhamis, et al., 2021).

Albeit other predictors in the best‐fit ERGMs had statistically significant effects on the transmission networks, interpreting those in terms of mode of transmission is challenging. High pig farm density has been underlined as a risk factor for PRRSV spread and persistence in several studies (Arruda, Vilalta, et al., 2017; Jara et al., 2021; Makau, Alkhamis, et al., 2021). Our model suggests that a pair of farms located in areas with similar density are more likely to transmit the virus to one another. Though it is unclear, one plausible explanation is if farm density represents an aspect of biosecurity investment. Locations in low‐density areas are often chosen for farms where biosecurity may be of particular importance, such as nucleus, multipliers, breeding herds, and boar studs, whereas there is often less investment in biosecurity at farms in higher‐density areas. Exclusively for cluster A, farm pairs sampling the virus in different seasons were significantly associated with the transmission link. This could be perhaps a function of the time of year (fall to winter) when disease incidence was primarily increasing (Trevisan et al., 2020). That being said, we do not have a good explanation for all the associations documented by our model, and it perhaps may be an artifact of an undocumented confounding factor.

We also found that transmission links were more likely if the time interval between samples was shorter. We included this factor to account for the temporality in epidemiological process. However, the results also provide insights into the farm‐level generation time (i.e., the lapse of time between the primary and a secondary farm involved in a transmission pair), assuming that sample collection dates are at least somewhat aligned to disease detection dates. The results of the ERGM suggest that the likelihood of such onward transmission is drastically reduced after approximately 2 months, even though the average sow farm takes 6–10 months to bring an outbreak under control (Linhares et al., 2014; Sanhueza et al., 2019). This would make intuitive sense if between‐farm transmission is correlated with prevalence or shedding on a farm, both of which would likely decrease after the initial acute phase of an outbreak. Such information may be useful in managing and mitigating the risk posed to other farms by farms experiencing PRRSV‐2 outbreaks.

While these findings provide a unique window into the dynamics of between‐farm PRRSV‐2 transmission, one limitation of the analysis is that it focused on the voluntarily submitted ORF5 gene of a specific sub‐lineage within a single, albeit large, swine‐producing region in the U.S., and we do not know the extent to which results can be generalized to other circulating PRRSV‐2 variants or to other regions or countries that may have distinct farming practices. In addition, although we cannot fully elucidate PRRSV‐2 evolutionary dynamics without full‐length genomes, genetic variation and phylogenies estimated from the ORF5 gene can yield comparable results when compared to genomic‐level analysis (Frias‐De‐Diego et al., 2021) and constraining the analysis to highly related genetic variants (>98% pairwise identity) decreases the likelihood that evolutionary analyses would be confounded by recombination.

Although this study fully focused on PRRS epidemiology, the approach we designed can be applied to assess other infectious disease transmissions comparable to our context. Swine pathogens with genetic marker that represents their evolution, such as HA and NA genes of swine influenza virus, spike (S) gene of porcine epidemic diarrhea virus, or whole genome sequence of African swine fever virus, likely fit with our analysis since they are or will be circulating in the same farming system as PRRSV‐2. Our principle that estimates the transmission between units (farms) comprising a group of individuals (animals) can also be extended to analyze the potential transmission risks of other livestock or even human diseases if the unit, between‐unit connection (animal movement in our case), and transmission routes are clearly defined.

Although the primary focus of this study was on PRRS epidemiology, the approach we devised has broader applicability and can be effectively employed to assess comparable infectious disease transmissions in similar contexts. Swine pathogens harboring genetic markers indicative of their evolution, such as the HA and NA genes of the swine influenza virus, the spike (S) gene of the porcine epidemic diarrhea virus, or the whole genome of the African swine fever virus, are likely compatible with our analytical framework, as they either circulate or are anticipated to emerge within the same farming system as PRRSV‐2. Moreover, our fundamental principle, which estimates transmission between units (farms) comprising groups of individuals (animals), can be readily extended to evaluate the potential transmission risks of other livestock diseases or even human diseases, provided that the units, between‐unit connections (as exemplified by animal movement in our case), and transmission routes are clearly defined.

## 5 CONCLUSION

This study reveals a clearer picture of regional PRRSV‐2 spread in the U.S. by bridging viral genetic data and animal movement data to infer farm‐to‐farm transmission events and associated risk factors. Each step of our approach highlights different epidemiological aspects. Phylodynamic analysis of each distinct cluster suggests that the regional PRRSV‐2 lineage L1A outbreak was characterized by multiple micro‐epidemics driven by different variants with temporally variable viral population sizes, which is consistent with the numerical frequency of sequences caused by each variant. The inferred farm‐to‐farm transmission network suggests that most infected farms spread the virus to an average of one other farm, which is expected for an endemic disease, but there were also several potential super‐spreader events (identified by the heterogeneity in individual farm's reproduction number). These super‐spreader events occurred at times with increasing effective population size shown by phylodynamic analysis and disease incidence indicated by frequency of sample submission. Animal movement data did not explain over 80% of the total transmission events. There was putative evidence for transmission from boar studs to sow farms via semen in some cases, but a purported route of transmission could not be determined for most events. This is unsurprising due to the paucity of data on between‐farm movement of people, trucks, and other equipment that likely can carry fomites. Animal movement, however, was indeed an important factor shaping the transmission network in that pairs of farms connected via animal movement were more likely to have a transmission link than pairs that were not. In contrast, farm proximity‐related factors, including airborne spread, did not appear to play a major role in shaping transmission networks according to the network analysis. Routine PRRS monitoring or testing, along with risk communication within and between the production systems, is necessary to prevent potential transmission links. Ultimately, this study not only provides new insights on PRRSV‐2 transmission dynamics in the U.S. but also initiates integrative approaches that may help improve livestock disease monitoring and surveillance.

## AUTHOR CONTRIBUTIONS

NP and KV conceived and designed the study. CC provided data for the analysis. IP and DM curated the data. KV supervised the direction of the analysis. NP performed data analysis and wrote the first draft of the paper. All authors have reviewed and approved the submitted version of the manuscript.

## FUNDING INFORMATION

This project was funded by the joint NIFA‐NSF‐NIH Ecology and Evolution of Infectious Disease award 2019–67015‐29918, the University of Minnesota College of Veterinary Medicine Signature Programs, grant number MIN‐62‐133, and the Critical Agricultural Research and Extension Program, grant number 2018–68008‐27890. NP was supported by the Royal Thai Government Scholarship. This work was partly funded by the University of Minnesota Swine Disease Eradication Center (SDEC) and the Swine Health Information Center (SHIC) as the funding agency for MSHMP.

## CONFLICT OF INTEREST STATEMENT

The authors declare no conflict of interest.

## DATA ACCESSIBILITY AND BENEFIT‐SHARING STATEMENT

RNA sequences were sub‐sampled from GenBank accessions MN498289–MN502669.

## Supporting information


**Data S1:** Supporting Information.

## Data Availability

The non‐sensitive example dataset presented in this study can be found in online repository at: https://github.com/NakarinP/FarmR.
