# Supplementary material for: Unveiling invisible farm‐to‐farm PRRSV‐2 transmission links and routes through transmission tree and network analysis
Source: Evol Appl. 2023 Sep 15;16(10):1721–34. doi: 10.1111/eva.13596 (PMC10660809; doi:10.1111/eva.13596)
Supplement: Supplementary file 1 — Data S1: Supporting Information. [file EVA-16-1721-s001.docx]

**Supplementary Information**

***Supplementary table 1*** *Multivariable ERGMs’ covariates of farm-to-farm transmission network of cluster A*

| Model | Covariates | df | AIC |
| --- | --- | --- | --- |
| mod23 | In-stars + Movement pathlength + Distance between farms + Farm density (difference) + Time length + Sampling season (homophily) + Farm type (degree effect) | 8 | 639.39 |
| mod21* | In-stars + Movement pathlength + Distance between farms + Farm density (difference) + Time length + Sampling season (homophily) | 6 | 640.03 |
| mod22 | In-stars + Movement pathlength + Distance between farms + Farm density (difference) + Time length + Sampling season (homophily) + Movement network out-degree (degree effect) | 7 | 640.71 |
| mod26 | In-stars + Movement pathlength + Distance between farms + Farm density (difference) + Time length + Sampling season (homophily) + Farm type (degree effect) + Herd size (degree effect) | 9 | 641.16 |
| mod27 | In-stars + Movement pathlength + Distance between farms + Farm density (difference) + Time length + Sampling season (homophily) + Farm type (degree effect) + Movement network in-degree (degree effect) | 9 | 641.24 |
| mod24 | In-stars + Movement pathlength + Distance between farms + Farm density (difference) + Time length + Sampling season (homophily) + Farm type (degree effect) + Farm density (degree effect) | 9 | 641.30 |
| mod28 | In-stars + Movement pathlength + Distance between farms + Farm density (difference) + Time length + Sampling season (homophily) + Farm type (degree effect) + Herd size (difference) | 9 | 641.30 |
| mod25 | In-stars + Movement pathlength + Distance between farms + Farm density (difference) + Time length + Sampling season (homophily) + Farm type (degree effect) + Farm type (homophily) | 9 | 641.36 |
| mod20 | In-stars + Movement pathlength + Distance between farms + Farm density (difference) + Time length | 5 | 642.26 |
| mod30 | In-stars + Movement pathlength + Distance between farms + Farm density (difference) + Time length + Sampling season (homophily) + Farm type (degree effect) + Farm type (heterophily) | 17 | 643.01 |
| mod29 | In-stars + Movement pathlength + Distance between farms + Farm density (difference) + Time length + Sampling season (homophily) + Farm type (degree effect) + Sampling season (degree effect) | 11 | 643.87 |
| mod19 | In-stars + Movement pathlength + Distance between farms + Farm density (difference) | 4 | 678.91 |
| mod18 | In-stars + Movement pathlength + Distance between farms | 3 | 681.58 |
| mod15.3 | In-stars + Movement pathlength | 2 | 696.30 |
| mod17 | In-stars + Distance between farms | 2 | 708.74 |
| mod11 | In-stars + Farm density (difference) | 2 | 728.69 |
| mod16 | In-stars + Time length | 2 | 729.58 |
| mod1 | In-stars | 1 | 729.83 |
| mod7 | In-stars + Sampling season (homophily) | 2 | 730.23 |
| mod14 | In-stars + Movement network out-degree (degree effect) | 2 | 731.31 |
| mod5 | In-stars + Farm type (degree effect) | 3 | 731.47 |
| mod12 | In-stars + Farm density (degree effect) | 2 | 731.59 |
| mod4 | In-stars + Farm type (homophily) | 2 | 731.65 |
| mod10 | In-stars + Herd size (degree effect) | 2 | 731.74 |
| mod13 | In-stars + Movement network in-degree (degree effect) | 2 | 731.75 |
| mod9 | In-stars + Herd size (difference) | 2 | 731.83 |
| mod8 | In-stars + Sampling season (degree effect) | 4 | 735.21 |
| mod3 | In-stars + Farm type (heterophily) | 10 | 740.63 |
| mod2 | k-Outstars | 1 | 1136.04 |
| mod15.1 | In-stars + Movement pathlength | 3 | 2647.73 |
| mod15.2 | In-stars + Movement pathlength | 2 | 3540.01 |
| mod6 | In-stars + Sampling season (heterophily) | 16 | NaN |
| mod31 | In-stars + Movement pathlength + Distance between farms + Farm density (difference) + Time length + Sampling season (homophily) + Farm type (degree effect) + Sampling season (heterophily) | 23 | NaN |

*The parsimonious (best-fit) model selected for interpretation

***Supplementary table 2*** *Multivariable ERGMs’ covariates of farm-to-farm transmission network of cluster B*

| Model | Covariates | df | AIC |
| --- | --- | --- | --- |
| mod26 | In-stars + Movement pathlength + Distance between farms + Farm density (difference) + Time length + Sampling season (homophily) + Farm type (degree effect) + Herd size (degree effect) | 9 | 293.02 |
| mod23 | In-stars + Movement pathlength + Distance between farms + Farm density (difference) + Time length + Sampling season (homophily) + Farm type (degree effect) | 8 | 293.49 |
| mod21* | In-stars + Movement pathlength + Distance between farms + Farm density (difference) + Time length + Sampling season (homophily) | 6 | 293.56 |
| mod36 | In-stars + Movement pathlength + Distance between farms + Farm density (difference) + Time length + Sampling season (homophily) + Farm type (degree effect) + Herd size (degree effect) + Farm type (homophily) | 10 | 294.83 |
| mod22 | In-stars + Movement pathlength + Distance between farms + Farm density (difference) + Time length + Sampling season (homophily) + Movement network out-degree (degree effect) | 7 | 294.83 |
| mod34 | In-stars + Movement pathlength + Distance between farms + Farm density (difference) + Time length + Sampling season (homophily) + Farm type (degree effect) + Herd size (degree effect) + Movement network in-degree (degree effect) | 10 | 294.98 |
| mod35 | In-stars + Movement pathlength + Distance between farms + Farm density (difference) + Time length + Sampling season (homophily) + Farm type (degree effect) + Herd size (degree effect) + Herd size (difference) | 10 | 294.99 |
| mod33 | In-stars + Movement pathlength + Distance between farms + Farm density (difference) + Time length + Sampling season (homophily) + Farm density (degree effect) | 7 | 295.53 |
| mod37 | In-stars + Movement pathlength + Distance between farms + Farm density (difference) + Time length + Sampling season (homophily) + Farm type (degree effect) + Herd size (degree effect) + Sampling season (degree effect) | 12 | 298.76 |
| mod32 | In-stars + Movement pathlength + Distance between farms + Farm density (difference) + Sampling season (homophily) | 5 | 318.33 |
| mod19 | In-stars + Movement pathlength + Distance between farms + Farm density (difference) | 4 | 324.14 |
| mod18 | In-stars + Movement pathlength + Distance between farms | 3 | 329.69 |
| mod15.3 | In-stars + Movement pathlength | 2 | 334.96 |
| mod15.2 | In-stars + Movement pathlength | 2 | 339.80 |
| mod17 | In-stars + Distance between farms | 2 | 346.01 |
| mod11 | In-stars + Farm density (difference) | 2 | 349.01 |
| mod7 | In-stars + Sampling season (homophily) | 2 | 351.58 |
| mod16 | In-stars + Time length | 2 | 351.67 |
| mod14 | In-stars + Movement network out-degree (degree effect) | 2 | 354.69 |
| mod1 | In-stars | 1 | 354.74 |
| mod12 | In-stars + Farm density (degree effect) | 2 | 354.88 |
| mod5 | In-stars + Farm type (degree effect) | 3 | 355.10 |
| mod10 | In-stars + Herd size (degree effect) | 2 | 356.47 |
| mod13 | In-stars + Movement network in-degree (degree effect) | 2 | 356.53 |
| mod9 | In-stars + Herd size (difference) | 2 | 356.68 |
| mod4 | In-stars + Farm type (homophily) | 2 | 356.69 |
| mod8 | In-stars + Sampling season (degree effect) | 4 | 359.31 |
| mod15.1 | In-stars + Movement pathlength | 3 | 391.18 |
| mod2 | k-Outstars | 1 | 518.96 |
| mod3 | In-stars + Farm type (heterophily) | 7 | NaN |
| mod6 | In-stars + Sampling season (heterophily) | 12 | NaN |
| mod38 | In-stars + Movement pathlength + Distance between farms + Farm density (difference) + Time length + Sampling season (homophily) + Farm type (degree effect) + Herd size (degree effect) + Farm type (heterophily) | 15 | NaN |
| mod39 | In-stars + Movement pathlength + Distance between farms + Farm density (difference) + Time length + Sampling season (homophily) + Farm type (degree effect) + Herd size (degree effect) + Sampling season (heterophily) | 20 | NaN |

*The parsimonious (best-fit) model selected for interpretation

***Supplementary table 3*** *Multivariable ERGMs’ covariates of farm-to-farm transmission network of cluster C*

| Model | Covariates | df | AIC |
| --- | --- | --- | --- |
| mod49 | In-stars + Distance between farms + Farm type (homophily) + Movement pathlength + Farm density (difference) + Time length + Movement network in-degree (degree effect) | 7 | 350.85 |
| mod48* | In-stars + Distance between farms + Farm type (homophily) + Movement pathlength + Farm density (difference) + Time length | 6 | 350.89 |
| mod50 | In-stars + Distance between farms + Farm type (homophily) + Movement pathlength + Farm density (difference) + Time length + Movement network in-degree (degree effect) + Farm type (degree effect) | 9 | 353.57 |
| mod51 | In-stars + Distance between farms + Farm type (homophily) + Movement pathlength + Farm density (difference) + Time length + Movement network in-degree (degree effect) + Sampling season (degree effect) | 10 | 354.76 |
| mod53 | In-stars + Distance between farms + Farm type (homophily) + Movement pathlength + Farm density (difference) + Time length + Movement network in-degree (degree effect) + Farm type (heterophily) | 16 | 365.10 |
| mod52 | In-stars + Distance between farms + Farm type (homophily) + Movement pathlength + Farm density (difference) + Time length + Movement network in-degree (degree effect) + Sampling season (heterophily) | 23 | 368.43 |
| mod42 | In-stars + Distance between farms + Farm type (homophily) + Movement pathlength + Farm density (difference) | 5 | 369.08 |
| mod47 | In-stars + Distance between farms + Farm type (homophily) + Movement pathlength + Farm density (difference) + Farm density (degree effect) | 6 | 369.17 |
| mod41 | In-stars + Distance between farms + Farm type (homophily) + Movement pathlength | 4 | 370.10 |
| mod40 | In-stars + Distance between farms + Farm type (homophily) | 3 | 370.20 |
| mod44 | In-stars + Distance between farms + Farm type (homophily) + Movement pathlength + Farm density (difference) + Sampling season (homophily) | 6 | 370.54 |
| mod43 | In-stars + Distance between farms + Farm type (homophily) + Movement pathlength + Farm density (difference) + Herd size (difference) | 6 | 370.82 |
| mod46 | In-stars + Distance between farms + Farm type (homophily) + Movement pathlength + Farm density (difference) + Movement network out-degree (degree effect) | 6 | 370.84 |
| mod45 | In-stars + Distance between farms + Farm type (homophily) + Movement pathlength + Farm density (difference) + Herd size (degree effect) | 6 | 371.08 |
| mod17 | In-stars + Distance between farms | 2 | 375.02 |
| mod4 | In-stars + Farm type (homophily) | 2 | 404.96 |
| mod15.3 | In-stars + Movement pathlength | 2 | 407.37 |
| mod1 | In-stars | 1 | 408.37 |
| mod15.2 | In-stars + Movement pathlength | 2 | 408.39 |
| mod11 | In-stars + Farm density (difference) | 2 | 408.76 |
| mod9 | In-stars + Herd size (difference) | 2 | 408.95 |
| mod7 | In-stars + Sampling season (homophily) | 2 | 409.99 |
| mod10 | In-stars + Herd size (degree effect) | 2 | 410.03 |
| mod14 | In-stars + Movement network out-degree (degree effect) | 2 | 410.09 |
| mod12 | In-stars + Farm density (degree effect) | 2 | 410.24 |
| mod16 | In-stars + Time length | 2 | 410.26 |
| mod13 | In-stars + Movement network in-degree (degree effect) | 2 | 410.35 |
| mod5 | In-stars + Farm type (degree effect) | 3 | 412.33 |
| mod8 | In-stars + Sampling season (degree effect) | 4 | 412.43 |
| mod6 | In-stars + Sampling season (heterophily) | 17 | 417.57 |
| mod3 | In-stars + Farm type (heterophily) | 10 | 417.77 |
| mod2 | k-Outstars | 1 | 603.16 |
| mod15.1 | In-stars + Movement pathlength | 3 | 643.37 |

*The parsimonious (best-fit) model selected for interpretation


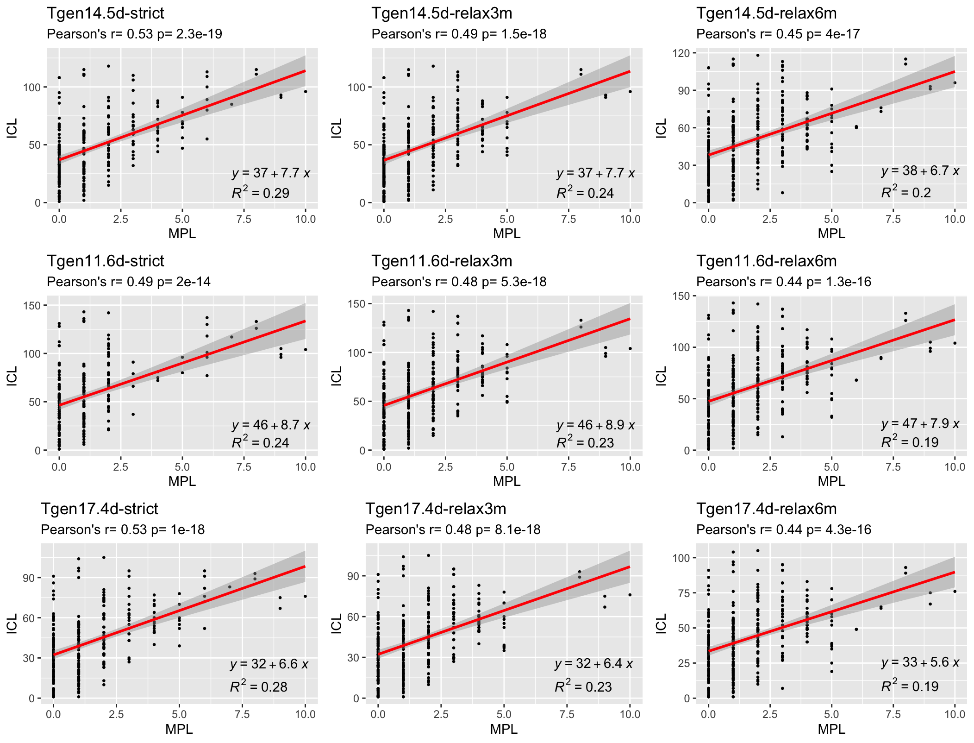


***Supplementary figure 1*** *Sensitivity analysis of the correlation between infection chain length (ICL) and movement pathlength (MPL) of all clusters varied by PRRS mean generation time (T_gen_); 11.6, 14.5, and 17.4 days, and the time frame for capturing matched animal movement events; strict (onset to terminus of an inferred infection chain), relax3m (3 months prior to the onset to terminus), relax6m (6 months prior to the onset to terminus).*


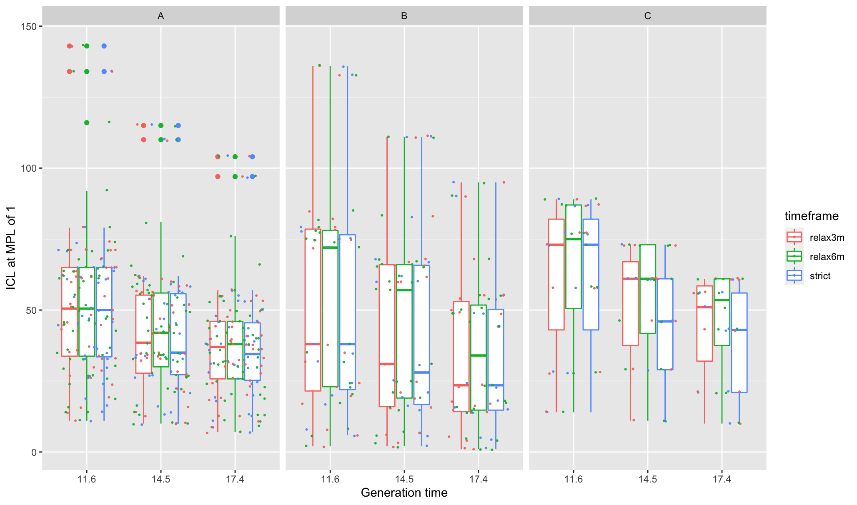


***Supplementary figure 2*** *Infection chain length (ICL) at movement pathlength (MPL) of 1 of each cluster (A to C) varied by PRRS mean generation time (T_gen_); 11.6, 14.5, and 17.4 days, and the time frame for capturing matched animal movement events; strict (onset to terminus of an inferred infection chain), relax3m (3 months prior to the onset to terminus), relax6m (6 months prior to the onset to terminus).*

**
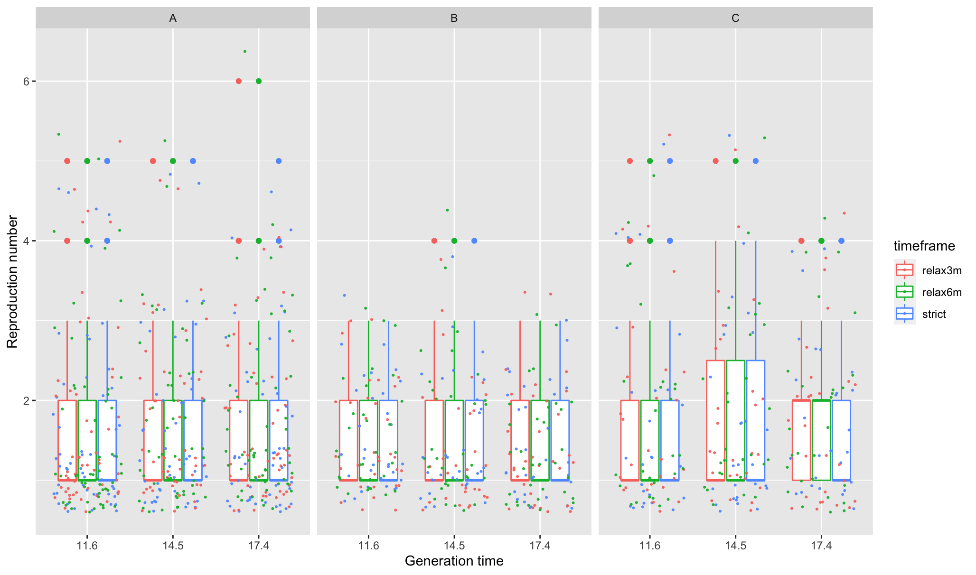
**

***Supplementary figure 3*** *Farm-level effective reproduction number (R) of each cluster (A to C) varied by PRRS mean generation time (T_gen_); 11.6, 14.5, and 17.4 days, and the time frame for capturing matched animal movement events; strict (onset to terminus of an inferred infection chain), relax3m (3 months prior to the onset to terminus), relax6m (6 months prior to the onset to terminus).*


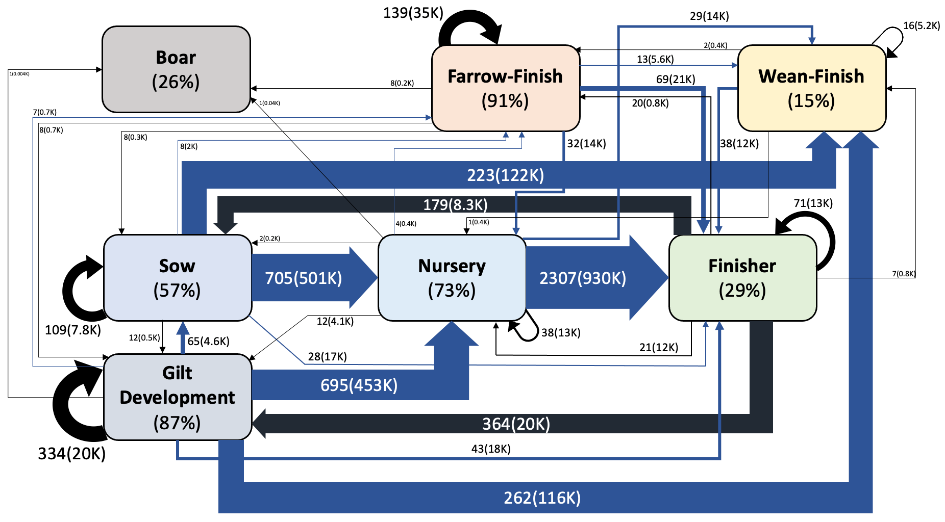


***Supplement figure 4*** *Monthly animal movement among pig sites in the study production system from January 2014 to December 2017. (Box) The number in parentheses beneath production type represents the mean proportion of active sites. (Arrow) The arrow thickness and the number within the arrow demonstrate the mean number of monthly shipments with the mean number of pigs in parentheses. The directions of movement in comparison to the production flow are represented by arrow’s color (Blue; toward the flow, Black; against the flow or movement to the same production type)*
